# The relationship between phospholipids and insulin resistance: From clinical to experimental studies

**DOI:** 10.1111/jcmm.13984

**Published:** 2018-11-06

**Authors:** Wenguang Chang, Grant M. Hatch, Yu Wang, Fei Yu, Man Wang

**Affiliations:** ^1^ Center for Regenerative Medicine Institute for Translational Medicine Qingdao University Qingdao China; ^2^ Departments of Pharmacology and Therapeutics, Biochemistry and Medical Genetics Center for Research and Treatment of Atherosclerosis DREAM Children's Hospital Research Institute of Manitoba University of Manitoba Winnipeg MB Canada

**Keywords:** glycerolphospholipid, insulin resistance, insulin sensitivity, phospholipids

## Abstract

Insulin resistance induced by high‐fat diet and impropriate life style is a major contributor to the pathogenesis of metabolic disease. However, the underlying molecular mechanisms remain unclear. Recent studies in metabolic dysfunction have extended this beyond simply elevated cholesterol and triglycerides levels and have identified a key role for lipid metabolism. For example, altered phospholipid metabolism has now become central in the pathogenesis of metabolic disease. In this review, we discuss the association between insulin sensitivity and phospholipid metabolism and highlight the most significant discoveries generated over the last several decades. Finally, we summarize the current knowledge surrounding the molecular mechanisms related to phospholipids and insulin resistance and provide new insight for future research into their relationship.

## 1 INTRODUCTION

Obesity, insulin resistance, type 2 diabetes mellitus and macrovascular diseases are main culprits in the development of metabolic diseases.^1^, ^2^ According to the International Diabetes Federation, the population of obese individuals has increased dramatically in developing countries over the past two decades and the number of people with diabetes mellitus is projected to rise to 592 million globally by 2035.^3^ Insulin resistance is the most common pathological factor accompanied by obesity and type 2 diabetes. The presence of insulin resistance in insulin‐sensitive target tissues results in major abnormalities such as hyperglycaemia, hyperinsulinemia and hypertriglyceridemia which are common features of the metabolic syndrome.^2^, ^4^ For decades, high triacylglycerol and cholesterol levels were considered to be the cause of this metabolic disease,^5^, ^6^, ^7^ However, recent studies have indicated that in addition to the raised triacylglycerol and cholesterol levels, phospholipid alterations may also play a role in the pathological process of metabolic disorders.^8^, ^9^, ^10^, ^11^ In this review, we assemble recent clinical and experimental evidence for the role and mechanism of phospholipids in the pathogenesis of insulin resistance. These studies aid us in our understanding of the complex biology of metabolic disorders and provide a new insight for further research into the biology of insulin resistance.

## 2 PHOSPHOLIPID SYNTHESIS

**Figure 1:**
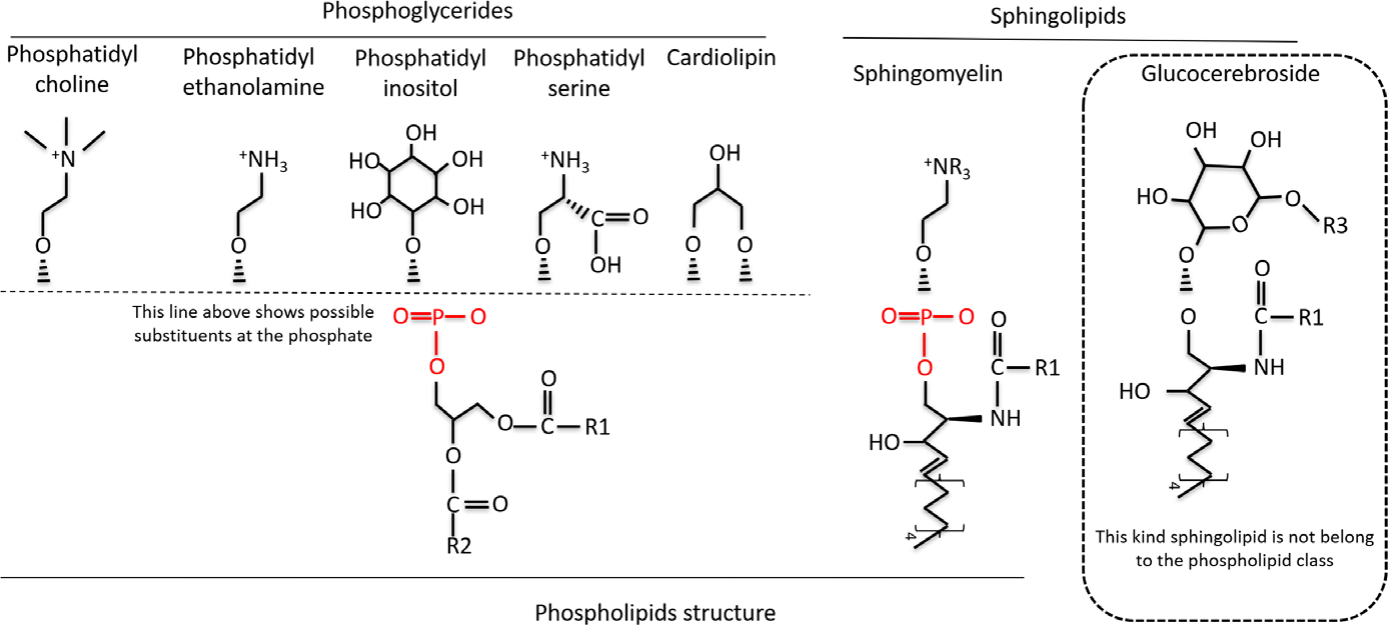
Structures of phospholipids

**Figure 2:**
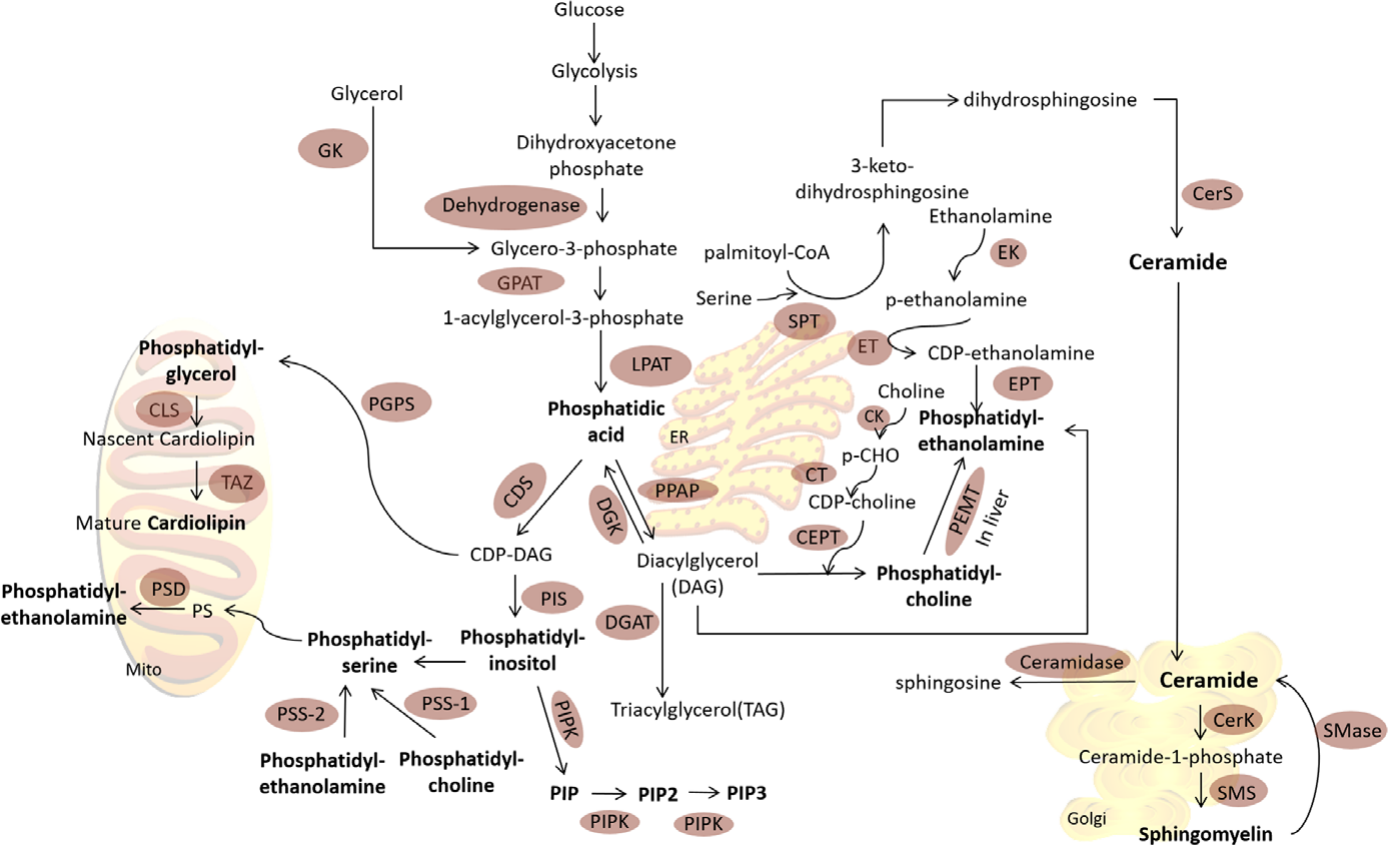
Biosynthetic pathways for phospholipids in mammalian cells

Phospholipids are a class of lipids that represent a major component of the bilayer of cell membranes.^12^, ^13^ The structure of the phospholipid molecule generally consists of two hydrophobic fatty acid “tails” and a hydrophilic “head” comprising a phosphate group. They may also be divided into two groups through differences in their backbones. One group are the glycerol‐based phospholipids (glycerophospholipids). The phosphate groups are modified with simple organic molecules such as choline (to form phosphatidylcholine), ethanolamine (to form phosphatidylethanolamine), inositol (to form phosphatidylinositol), serine (to form phosphatidylserine) or two phosphatidic acid moieties connected with a glycerol backbone in the centre to form cardiolipin (Figure 1). The other is a class of lipids containing the backbone of a sphingosine base, sphingolipids^14^ (Figure 1). Only sphingomyelin, which contains a phosphate group, belongs to this group of phospholipids. In mammalian cells, de novo synthesis of glycerophospholipids requires the acquisition of diacylglycerol units obtained through either diacylglycerol or cytidine diphosphate‐diacylglycerol (CDP‐DAG) synthesized from phosphatidic acid^15^, ^16^ (Figure 2). In the early de novo synthesis steps, glycerol is converted to glycerol‐3‐phosphate by glycerol kinase (GK). In addition, dihydroxyacetonephosphate generated from glucose through glycolysis is converted to glycerol‐3‐phosphate by glycerol‐3‐phosphate dehydrogenase. Then glycerol phosphate acyltransferase converts glycerol‐3‐phosphate into 1‐acylglycerol‐3‐phosphate. 1‐Acylglycerol‐3‐phosphate is converted to phosphatidic acid through the action of lysophosphatidic acid acyltransferase, acyltransferases associated with both the endoplasmic reticulum (ER) and outer membrane of mitochondria.^17^ Finally, diacylglycerol and CDP‐DAG are generated from phosphatidic acid catalysed by phosphatidic acid phosphatase or CDP‐diacylglycerol synthetase, respectively, which are also associated primarily with ER and mitochondrial membranes.^18^, ^19^ Diacylglycerol is incorporated into phosphatidylcholine, and phosphatidylethanolamine (Figure 2). Phosphatidylcholine was the first phospholipid identified in biological tissues and rich sources are found in the egg yolk of chickens. Phosphatidylcholine is synthesis via cytidine diphosphate‐choline pathway (CDP‐choline), also known as the Kennedy pathway.^20^, ^21^ In brief, extracellular choline is imported into the cell and rapidly phosphorylated to phosphocholine by the cytosolic enzyme choline kinase. Phosphocholine is then converted to CDP‐choline by membrane CTP:phosphocholine cytidylyltransferase (CT), the rate‐limiting reaction for phosphatidylcholine biosynthesis.^22^, ^23^ Finally, 1,2‐diacylglycerol cholinephosphotransferase catalyses the formation of phosphatidylcholine from CDP‐choline and diacylglycerol. In a similar manner, phosphatidylethanolamine is also made in the ER by the CDP‐ethanolamine pathway.^24^ Ethanolamine is first phosphorylated to phosphoethanolamine by ethanolamine kinase and then converted to CDP‐ethanolamine by a cytosolic protein CTP:phosphoethanolamine cytidylyltransferase (ET, Pcyt2). Subsequently, the ER integral membrane protein 1,2‐diacylglycerolethanolamine phosphotransferase (EPT) catalyses formation of phosphatidylethanolamine from CDP‐ethanolamine and diacylglycerol.^23^ Phosphatidylserine is synthesized by a base‐exchange reaction from either phosphatidylcholine (via phosphatidylserine synthase‐1) or phosphatidylethanolamine (via phosphatidylserine synthase‐2) (Figure 2). These enzymatic reactions occur in the ER.^25^, ^26^ Alternatively, in mitochondria, phosphatidylethanolamine may also be synthesized by decarboxylation of phosphatidylserine catalysed by phosphatidylserine decarboxylase, an enzyme restricted to mitochondrial inner membranes.^26^ Unique to the mammalian liver, phosphatidylethanolamine may also be converted to phosphatidylcholine by phosphatidylethanolamine *N*‐methyltransferase (PEMT) in mitochondrial‐associated membranes (MAM). PEMT activity has proven to be critical for insulin sensitivity.^24^, ^27^ In addition to diacylglycerol, CDP‐DAG participates in the synthesis of phosphatidylinositol and its numerous phosphorylated derivatives that are involved in cell signalling events.^28^ Phosphatidylinositol is synthesized by phosphatidylinositol synthase in the ER from *myo*‐inositol and CDP‐diacylglycerol (Figure 2). Once synthesized, phosphatidylinositol can be phosphorylated by phosphatidylinositol phosphate kinases (PIPK, including phosphatidylinositide 3‐kinase, PI3K; phosphatidylinositide 4‐kinase), which catalyse the addition of a phosphate group to 3′‐ or 4′‐ or 5′‐positions of the inositol ring of phosphatidylinositol to form seven molecular species, including PIP (phosphatidylinositol 3‐phosphate, phosphatidylinositol 4‐phosphate or phosphatidylinositol 5‐phosphate), PIP2 (phosphatidylinositol 3,4‐bisphosphate, phosphatidylinositol 3,5‐bisphosphate or phosphatidylinositol 4,5‐bisphosphate) and PIP3 (phosphatidylinositol 3,4,5‐triphosphate)^29^ (Figure 2). CDP‐DAG generated in mitochondria and ER form phosphatidylglycerolphosphate (PGP) through the action of PGP synthase. This is followed by dephosphorylation by phosphatidylglycerolphosphate phosphatase to form phosphatidylglycerol (PG). In mitochondria, PG combines with a second molecule of CDP‐DAG in a reaction catalysed by cardiolipin synthase to form cardiolipin. Cardiolipin may be remodelled by calcium‐independent phospholipase A_2_ hydrolysis followed by reacylation by acyltransferases, including monolysocardiolipin acyltransferase‐1 and through transacylation by the enzyme tafazzin.^30^, ^31^ One of these acyltransferases, ALCAT1, is localized to the MAM. In addition to glycerolphospholipids, sphingomyelin is synthesized in a pathway that begins in the ER and is completed in the Golgi apparatus. De novo sphingomyelin synthesis begins with formation of 3‐keto‐dihydrosphingosine catalysed by serine palmitoyltransferase. Next, 3‐keto‐dihydrosphingosine is reduced to form dihydrosphingosine. Dihydrosphingosine is acylated by (dihydro)‐ceramide synthase (CerS) to form dihydroceramide, then desaturated to form ceramide. Ceramide is subsequently phosphorylated by ceramide kinase (CerK) to form ceramide‐1‐phosphate. The ceramide‐1‐phosphate is then converted to sphingomyelin by the addition of a phosphorylcholine headgroup catalysed by sphingomyelin synthase (SMS). Finally, sphingomyelin may be degraded by a sphingomyelinase (SMase) to form ceramide, and the ceramide may be further degraded by ceramidase to form sphingosine (Figure 2). As a coordinated group, phospholipids maintain cell membrane integrity and provide a matrix for many essential biochemical reactions and physiological processes. Any disruption of phospholipid metabolism could result in an undermining of cell membrane integrity, eventually causing cell function loss and cell death.^32^, ^33^ Although the exact regulatory mechanisms that govern phospholipid metabolism are still unclear, drugs targeted to phospholipids have shown great promise in cancer treatment.^34^, ^35^, ^36^, ^37^ In the case of insulin sensitivity, phospholipid alterations are known to be associated with insulin resistance. However, whether this association is the cause of insulin resistance or simply a consequence requires further discussion.

## 3 CLINICAL EVIDENCE FOR THE ASSOCIATION BETWEEN PHOSPHOLIPIDS AND INSULIN SENSITIVITY

Since phosphatidylcholine and phosphatidylethanolamine are the most abundant phospholipids in mammalian cells and are easy to obtain from human blood and small biopsy tissues, clinical studies have focused mainly on the relationship between insulin sensitivity and phosphatidylcholine, phosphatidylethanolamine or fatty acid composition of phosphatidylcholine or phosphatidylethanolamine. Clinical studies have demonstrated that decreased insulin sensitivity was associated with decreased concentration of polyunsaturated fatty acids (PUFA) in skeletal muscle phospholipids.^38^ This suggested that changes in the fatty acid composition of the cell membranes may modulate the action of insulin. Subsequently, a study conducted in older adults (age >70) showed a positive relationship between the proportion of palmitic acid (C16:0) in the skeletal muscle phospholipids and the insulin sensitivity index.^39^ More recently, a small group study confirmed this association and further observed that the fatty acid composition of phosphatidylcholine, and not phosphatidylethanolamine, from skeletal muscle membranes is of particular importance in this relationship.^40^ In that study, healthy patients were treated with nicotinic acid, an agent known to induce insulin resistance in humans. Treatment with nicotinic acid was associated with a 25% increase in the half‐maximal insulin concentration and decreased peripheral insulin sensitivity, and this was accompanied by significant increase in the phosphatidylcholine (C16:0), decrease phosphatidylcholine (C18:0) and long‐chain n‐3 fatty acid and PUFA. Recently, a clinical study in elderly participants also showed an association between decreased odds of abnormal homoeostasis model assessment‐insulin resistance (HOMA‐IR) and some phosphatidylcholine species (C32:0, C32:1, C32:2, C34:1, C34:2, C34:3, C36:2, C36:3, C40:5, C40:6, C42:3, C42:4 and C42:5).^41^ The relationship between lysophosphatidylcholine species and insulin resistance has also been investigated. Lysophosphatidylcholine is a hydrolysis product of phosphatidylcholine catalysed by phospholipase A_2_. A small human cohort showed that plasma lysophosphatidylcholine concentrations were reduced in obese adults and those with type 2 diabetes.^42^ In addition, certain species of lysophosphatidylcholines, such as lysophosphatidylcholine (C18:2), were suggested to be an independent predictor for incidence of type 2 diabetes.^43^, ^44^ In a study of 4297 adults in the population‐based Cooperative Health Research in the Region of Augsburg (KORA) cohort, adults with low serum lysophosphatidylcholine (C18:2) had greater risk of developing impaired glucose tolerance over a 7‐year follow‐up period.^45^ In addition to alterations in fatty acid species, changes in the total content of phosphatidylcholine or phosphatidylethanolamine are also associated with insulin insensitivity. A clinical study conducted in lean control and overweight but non‐diabetic patients showed that baseline fasting plasma insulin and HOMA‐IR were positively correlated with erythrocyte membrane phosphatidylethanolamine and phosphatidylcholine content in the whole population.^9^ Another clinical study in obese patients, who underwent surgery‐induced weight loss, demonstrated that the mRNA level of choline/ethanolamine phosphotransferase 1 (a key protein in final step of phosphatidylcholine synthesis) was inversely correlated with insulin sensitivity.^46^ The ratio of phosphatidylcholine to phosphatidylethanolamine has also attracted much attention. One elegant study showed that in the basal state, skeletal muscle phosphatidylcholine:phosphatidylethanolamine ratio was elevated in type 2 diabetic patients compared with obese patients and endurance‐trained athletes.^47^ A correlation analysis indicated that basal phosphatidylcholine:phosphatidylethanolamine ratio was negatively related to insulin sensitivity among all participants.^47^ In the same study, all participants exercised on a cycle ergometer for 90 minutes followed by 90 minutes recovery. Although the phosphatidylcholine and phosphatidylethanolamine content had significant group specific changes (phosphatidylcholine and phosphatidylethanolamine reduced during recovery in endurance trained athletes; phosphatidylethanolamine increased during recovery in type 2 diabetic patients), the skeletal muscle ratio of phosphatidylcholine to phosphatidylethanolamine remained unchanged in all groups, suggesting a single session of exercise may not be sufficient to alter this ratio.

Other glycerolphospholipids, such as cardiolipin and phosphatidylinositol derivatives, have been shown to be associated with insulin sensitivity in clinical studies.^48^, ^49^ Cardiolipin content was increased by daily moderate‐intensity exercise in type 2 diabetic patients and this was accompanied by improved insulin sensitivity.^49^ Interestingly, obese patients with weight loss through gastric bypass surgery showed changes in only specific fatty acid species of cardiolipin after exercise without significant changes in total cardiolipin content.^50^ In that study, exercise increased insulin sensitivity compared to control was accompanied by increases in the cardiolipin (C18:2)_4_ species and decreased cardiolipin (C18:2)_3_ (C18:1)_1_, cardiolipin (C18:2)_2_ (C18:1)_2_ and cardiolipin (C18:2)_3_ (C18:0)_1_ species without significant changes in total cardiolipin content.

Defects in glucose transport and atypical protein kinase C (αPKC) activation were observed in cultured myocytes obtained from obese/impaired glucose tolerant humans.^51^ PIP3, which is a derivate of phosphatidylinositol by class I phosphoinositide 3‐kinases (PI3K) (Figure 2), is known to mediate insulin effects on glucose transport through αPKC.^48^ In this clinical hyperinsulinemic‐euglycemic clamp study, compared to control, insulin induced αPKC activation was diminished by decreased ability of PIP3 to directly activate αPKCs in the impaired glucose tolerance and type 2 diabetic groups. It was suggested that this defective αPKC activation, mediated by PIP3, may contribute to skeletal muscle insulin resistance.

In addition to glycerolphospholipids, altered sphingomyelin was shown to be associated with insulin resistance in clinical studies. Erythrocyte membrane and adipocyte membrane sphingomyelin content were positively correlated with baseline fasting plasma insulin and HOMA‐IR in lean and overweight (but non‐diabetic) participants.^9^, ^10^ A clinical study showed that some fatty acid species of sphingomyelin, such as sphingomyelin (C18:0) but not total sphingomyelin were positively correlated with insulin resistance,^52^ However, another clinical study found that sphingomyelin (C16:0, C24:1, C26:1) were associated with decreased odds of abnormal HOMA‐IR in older adults (average age>70),^41^ suggesting that individual sphingomyelin species may have differing relationships with insulin resistance.

Although clinical studies have shown the association between phospholipids and insulin sensitivity, it is still not clear whether changes in phospholipids are the cause or consequence of insulin resistance. Recently, a metabolomic analysis of 431 participants was conducted including 133 newly developed type 2 diabetics from 2117 normoglycemic American Indians followed for an average of 5.5 years,^11^ Phosphatidylcholine (22:6, 20:4) along with three other metabolites were significantly associated with decreased risk of diabetes,^11^ suggesting an abnormal composition of phosphatidylcholine may be a cause of insulin resistance. However, the interrelationship between insulin sensitivity and phospholipids is a very complex process as multiple cell signal changes are related to altered phospholipid metabolism in response to insulin stimulation.

## 4 EXPERIMENTAL EVIDENCE FOR THE ASSOCIATION BETWEEN PHOSPHOLIPIDS AND INSULIN SENSITIVITY AND ITS RELATED MECHANISMS

### 4.1 The association between phosphatidylcholine, phosphatidylethanolamine and insulin sensitivity

**Figure 3:**
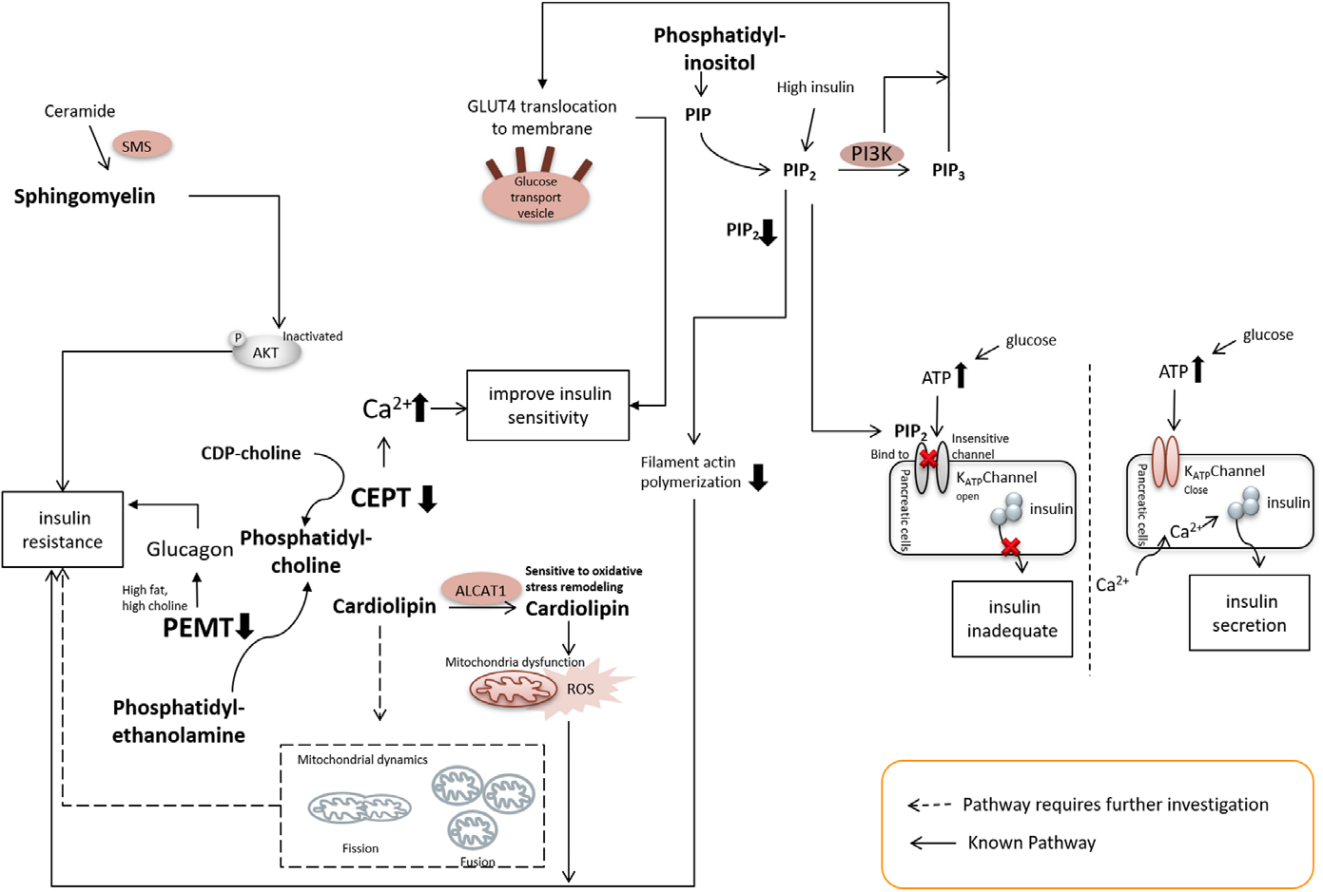
A summary of possible molecular mechanisms of phospholipids targeting insulin signalling

The relationship between insulin sensitivity and phosphatidylcholine or phosphatidylethanolamine content in experimental studies is vague. Whether changes in phosphatidylcholine and phosphatidylethanolamine content play a key role in insulin sensitivity is controversial. In a mouse model, reduced phosphatidylcholine levels mediated by hepatic specific knockout of CT, which is the key enzyme for phosphatidylcholine biosynthesis, were shown to lead to triacylglycerol accumulation but had no effect on insulin sensitivity.^53^ However, perturbed phosphatidylcholine synthesis through skeletal muscle specific knockdown of CEPT, the rate‐limiting enzyme in the major route for phosphatidylcholine production, resulted in improved insulin sensitivity in mice treated with a high‐fat diet.^46^ This influence on insulin sensitivity was thought to be related to an increase in activation of calcium‐signalling pathways via decreased sarco/ER Ca^2+^ ATPase‐dependent calcium uptake (Figure 3). Deletion of EPT, the rate‐limiting enzyme for phosphatidylethanolamine production, in mice is associated with increase in diacylglycerol.^54^ Diacylglycerol accumulation is known to cause insulin resistance in cells.^55^ In these mice, although intramyocellular and membrane‐associated diacylglycerol was markedly increased, insulin resistance was not observed.^54^ PEMT, which is the key enzyme for the conversion of phosphatidylethanolamine to phosphatidylcholine, is only quantitatively significant in the liver.^56^ Mice with global PEMT knockout showed reduced fatty liver but developed glucose and insulin intolerance when fed a high fat and high choline diet.^57^, ^58^ In that study, choline induced glucose and insulin intolerance in PEMT global knockout mice through modulating plasma glucagon and its action in liver. The authors suggested that increased plasma glucagon mediated by PEMT knockout during high choline and high‐fat diet may be responsible for the insulin resistance in these mice (Figure 3).

### 4.2 The role of phosphatidylinositol and derivatives of phosphatidylinositol in insulin sensitivity and in insulin secretion

Phosphatidylinositol and its derivatives make up only about 10%‐20% of all phospholipids. Although a minor quantitative portion they are important intracellular second messengers and participate in universal signalling entities in cells, including vesicular trafficking, lipid distribution and metabolism, ion channels, pumps, transporters, and endocytic and exocytic processes.^59^, ^60^, ^61^ Derangements in phosphatidylinositol and its derivatives are responsible for many human diseases ranging from genetic disorders to the most common cancers and metabolic diseases.^59^ Insulin treatment of fat and muscle cells results in a rapid increase in glucose transport by controlling the amount of glucose transporter‐4 (GLUT4) translocation from cytoplasm to plasma membrane. In the process of insulin‐stimulated glucose uptake, PIP, PIP2 and PIP3 all play key roles in integrating the insulin receptor‐issued signals with GLUT4 surface translocation and glucose transport activation.^61^ PI3K is the key enzyme for generating phosphatidylinositol species. For more comprehensive information on phosphatidylinositol biology and function the reader is referred to reviews 58, 59, 60. Impaired PI3K signalling has been suggested to be the cause of peripheral insulin resistance.^62^ Mice with deficient PI3K subunit (heterozygous for p110α or p110β) displayed impaired glucose metabolism (Figure 3).^63^, ^64^ In addition, exogenous PIP3 supplementation in L6 muscle cells increased both glucose uptake and glucose utilization and the effect was mediated by the activation of GLUT4.^65^

PIP2 also participates in the insulin secretion process. As plasma glucose rises, the ATP concentration increases and ADP concentration decreases, resulting in K(ATP) channel closure, membrane depolarization, Ca^2+^ influx and insulin release (Figure 3). PIP2 stimulates K (ATP) channels and decreases channel sensitivity to ATP inhibition by channel interaction.^66^ In cultured β‐cells, disruption of channel interactions with PIP2 by overexpressing PIP2‐insensitive channel subunits lead to membrane depolarization and elevated basal level insulin secretion at low glucose concentrations. In contrast, facilitation of channel interactions with PIP2 through upregulation of PIP2 levels decreases the ATP sensitivity of endogenous K (ATP) channels in INS‐1 cells resulting in reduced ability to secrete insulin in the presence of high glucose (Figure 3).^66^ Furthermore, PIP2 regulated filamentous actin (F‐actin) polymerization is important for insulin sensitivity. Hyperinsulinemia induced PIP2/F‐actin dysregulation resulted in insulin resistance in 3T3‐L1 adipocytes (Figure 3).^67^, ^68^ Interestingly, addition of PIP2 restored insulin responsiveness.^68^ Nevertheless, recent studies have demonstrated that the impaired metabolism of PIP3 is a prime mediator of insulin resistance associated with various metabolic diseases including obesity and diabetes.^69^ Above all, phosphatidylinositol and its derivatives have a close association with insulin sensitivity and disruption of PI metabolism may lead to insulin resistance.

### 4.3 The role of cardiolipin in mitochondrial function and insulin sensitivity

Mitochondria contain all the major classes of phospholipids.^70^, ^71^, ^72^ Unlike other abundant phospholipids, cardiolipin is exclusively localized to the mitochondria.^73^, ^74^ Most studies have focused on the role of cardiolipin in regulating mitochondrial function.^30^, ^31^, ^75^, ^76^, ^77^, ^78^ Changes in cardiolipin content as well as its fatty acid composition have been observed in models of diabetes. Dramatic loss of abundant cardiolipin molecular species were accompanied by a profoun remodelling of the remaining cardiolipin molecular species, including increase in the content of cardiolipin (18:2‐22:6‐22:6‐22:6), in the hearts of STZ‐treated mice at the very earliest stages of diabetes.^79^ Furthermore, it was shown that ALCAT1, a lysocardiolipin acyltransferase which is up‐regulated by oxidative stress and by diet‐induced obesity, preferentially catalysed the synthesis of cardiolipin species sensitive to oxidative stress in mice.^80^ This resulted in mitochondrial dysfunction with a higher production of reactive oxygen species leading to insulin resistance (Figure 3). Global ALCAT1 deficiency protected mice from diet‐induced obesity and insulin resistance.^80^ Mice with reduced cardiolipin mediated by knockdown of tafazzin, a transacylase required for cardiolipin remodelling (Figure 2), were resistant to high‐fat diet‐induced insulin desensitization.^81^

Cardiolipin also participates in mitochondrial dynamic regulation. Mitochondrial dynamic changes are closely related with insulin resistance. Mice with liver‐specific mitofusin 2 (Mfn2) knockdown (a key protein for mitochondria fusion) exhibited hepatic insulin resistance and glucose intolerance. Enhanced mitochondrial fission has been observed in the skeletal muscle of obese rodents and humans and this is accompanied by insulin resistance.^52^, ^82^, ^83^ Moreover, high‐fat feeding of rats induces mitochondrial fission and insulin resistance in dorsal vagal complex of brain via alterations in Drp1.^84^ Overexpression of ALCAT1, which increases long‐chain polyunsaturated fatty acids species of cardiolipin, resulted in reduction in the mitochondrial fusion essential protein Mfn2 and its mRNA expression.^85^ Conversely, knockdown ALCAT1 increased Mfn2 protein and its mRNA expression.^85^, ^86^ In addition, cardiolipin was shown to be required directly for activation of DRP1.^87^ For more detailed information with regard to cardiolipin and mitochondrial dynamics see Reference.^56^ Although there is limited direct evidence connecting cardiolipin to insulin sensitivity, it is possible that cardiolipin may regulate insulin resistance through modulation of mitochondrial dynamics (Figure 3).

### 4.4 The association of sphingomyelin with insulin sensitivity

Sphingolipids, including ceramides, sphingomyelins and gangliosides, are in low abundance relative to the levels of phosphatidylcholine or phosphatidylethanolamine in cells. As mentioned above, only sphingomyelin contains a phosphate group and belongs to the phospholipid class. Experimental studies have suggested that decreasing sphingomyelin may be associated with increasing insulin sensitivity. SMS is the terminal enzyme in sphingomyelin biosynthesis (Figure 2), and SMS2 is one of its isoforms. Global SMS2 deficiency in mice reduced liver sphingomyelin levels by 20%, and SMS2 deficiency prevented high‐fat diet‐induced obesity and insulin resistance.^88^ This observation was confirmed in HepG2 cells and it was further observed that AKT phosphorylation was decreased in a sphingomyelin concentration‐dependent manner.^89^ Thus, sphingomyelin may regulate insulin signalling through inactivation of AKT (Figure 3).^89^ In contrast, although global SMS2 knockout mice showed reduced insulin resistance induced by high‐fat diet, liver‐specific SMS2 knock out in mice had no effect on whole body insulin resistance, even though significant decreases in hepatic sphingomyelin were observed.^90^ In that study, genetic ablation of SMS2 elevated glucose clearance and this was attributed to activation of glucose uptake into insulin‐targeted tissues such as skeletal muscle.

## 5 CONCLUSIONS

Experimental and clinical studies have produced a large body of evidence implicating the role of phospholipids in a diverse range of physiological processes and as critical modulators of insulin sensitivity. In this review, we provided a brief view of each kind of phospholipid and their relationship with insulin resistance. Phospholipids influence insulin action in a number of ways, from regulating insulin secretion in pancreatic cells to mediating insulin action on skeletal muscle and adipocytes and modulating gene expression related glucose uptake as well as controlling mitochondrial dynamics (Figure 3). Although further investigation is still required in order to obtain more insight into the functional significance of these observations, phospholipids remain a promising target for controlling the physiological or pathophysiological processes affecting insulin sensitivity.

## CONFLICT OF INTEREST

The authors confirm that there are no conflicts of interest.
